# Repurposing Candidate Drugs to Prevent SARS-CoV-2: A PharmLines Test-Negative Case–Control Study

**DOI:** 10.3390/ph19060861

**Published:** 2026-05-29

**Authors:** Guiling Zhou, Nina Dael, Stefan Verweij, Spyros Balafas, Sumaira Mubarik, Jens Bos, Anna Maria Gerdina Pasmooij, Debbie van Baarle, Peter G. M. Mol, Geertruida H. de Bock, Eelko Hak, Lifelines Corona Research Initiative

**Affiliations:** 1 Unit of Pharmaco-Therapy, -Epidemiology and -Economics (PTEE), Department of Pharmacy, University of Groningen, 9713 AV Groningen, The Netherlands; n.dael@rug.nl (N.D.); s.verweij@cbg-meb.nl (S.V.); s.balafas@rug.nl (S.B.); s.mubarik@rug.nl (S.M.); h.j.bos@rug.nl (J.B.); e.hak@rug.nl (E.H.); 2 Dutch Medicines Evaluation Board, 3503 RG Utrecht, The Netherlands; am.pasmooij@cbg-meb.nl (A.M.G.P.); p.mol@cbg-meb.nl (P.G.M.M.); 3 Division of Pharmacoepidemiology and Clinical Pharmacology, Utrecht Institute for Pharmaceutical Sciences (UIPS), Utrecht University, 3508 TB Utrecht, The Netherlands; 4 Virology and Immunology Research Group, Department of Medical Microbiology and Infection Prevention, University Medical Center Groningen, University of Groningen, 9713 AV Groningen, The Netherlands; d.van.baarle@umcg.nl; 5 Department of Clinical Pharmacy and Pharmacology, University Medical Center Groningen, University of Groningen, 9700 RB Groningen, The Netherlands; 6 Department of Epidemiology, University Medical Center Groningen, University of Groningen, 9700 RB Groningen, The Netherlands; g.h.de.bock@umcg.nl

**Keywords:** COVID-19, repurposing drugs, SARS-CoV-2, COVID-19 vaccination, SARS-CoV-2 variant, test negative case control, pharmacoepidemiology

## Abstract

**Background:** The rapid emergence of immune-evasive SARS-CoV-2 variants necessitates the identification of accessible, low-cost prophylactic strategies. While drug repurposing offers a time-efficient alternative to novel drug development, clinical evidence for existing medications in the general population remains limited. The PharmLines Initiative provided us with unique data linkage for this study to assess the associations between 42 candidate drugs and COVID-19 infection. Potential effect modification by dominant SARS-CoV-2 strain and COVID-19 vaccination status was addressed. **Methods:** We conducted a test-negative case–control study using data from the Lifelines cohort and University of Groningen IADB.nl dispensing database. Cases were adults with self-reported reverse transcription polymerase chain reaction (RT-PCR) test results for SARS-CoV-2 and controls had only negative results. Cases and controls were matched in age, sex, and testing date. The 42 candidate drugs were identified through a systematic review of prior publications. The primary outcome was SARS-CoV-2 infection. We applied multivariable conditional logistic regression to estimate the associations, with subgroup analyses for variant and vaccination effects. Significance levels were corrected for multiple testing. **Results:** From November 2020 to October 2022, we included 2019 test-positive cases and 4089 matched test-negative controls with a mean age of 57 years and 67% female. After adjustments for confounders, none of the studied drugs were associated with SARS-CoV-2 infection. When stratified by SARS-CoV-2 variants, chronic use of calcium channel blockers (adjusted odds ratio 2.13; 95% CI 1.45–3.13), diuretics (2.23; 95% CI 1.50–3.32), and metformin (4.31; 95% CI 1.91–9.69) were associated with increased risks of original strain SARS-CoV-2 infection. No significant associations were found in the vaccination status subgroup analysis. **Conclusions:** Despite limited statistical power for some drugs, none of the studied drugs showed protective associations against SARS-CoV-2 infection. Antihypertensives and metformin were associated with increased risk. These findings do not support the off-label use of these drugs as COVID-19 prophylaxis in the general population.

## 1. Introduction

The coronavirus disease 2019 (COVID-19) pandemic caused by the novel severe acute respiratory syndrome coronavirus 2 (SARS-CoV-2) has posed unprecedented challenges for global public health. Vaccination remains the most effective method of preventing COVID-19 in healthy individuals. However, immunocompromised patients may have difficulty developing a sufficient immune response after vaccination, leaving them vulnerable to symptomatic infection and severe complications such as hospitalization and death [1]. In addition, vaccine accessibility remains severely limited in low-income countries. As of 14 August 2024, only 28% of the population in low-income countries had achieved full vaccination [2]. Therefore, pharmacological prophylaxis could provide additional protection for individuals with inadequate responses to vaccination, particularly in low-income countries.

Repurposed drugs are approved drugs that are being used or studied to treat diseases other than those for which they were originally approved. They offer several advantages, including well-established safety profiles, known pharmacokinetics, and shorter development timelines. Unlike monoclonal antibodies such as tixagevimab-cilgavimab (brand name Evusheld) [3] which can lose efficacy due to mutations in spike proteins [4], repurposed drugs often target host pathways rather than specific viral components, making them less susceptible to viral mutations. In addition, compared to novel antivirals such as remdesivir, repurposed drugs tend to be more affordable and widely available [5], increasing their appeal, especially in resource-limited settings or during public health emergencies.

Numerous clinical trials and observational studies have explored the potential of various drugs proposed for repurposing in the context of COVID-19. While much of the existing research focuses on these drugs as treatments in hospital settings [6], their effectiveness as prophylactic agents against SARS-CoV-2 at the community level remains largely unknown. We previously reported on a systematic review and showed that effect sizes of studied drugs proposed for repurposing were inconsistent across studies and often derived from studies of low methodological quality [7]. Recommendations from the World Health Organization (WHO) and the European Medicines Agency (EMA) against using hydroxychloroquine (HCQ) [8,9] and ivermectin [10] for SARS-CoV-2 prevention are already 3 years old. In addition, traditional observational designs such as cohort and case–control studies are susceptible to healthcare-seeking behavior bias—where differences in the likelihood of seeking medical care can affect the detection of outcomes—which may confound the association between repurposed drugs and COVID-19 outcomes. Importantly, existing studies have not assessed whether the association of repurposed drugs varies across SARS-CoV-2 variants or whether COVID-19 vaccination status influences the association.

The significant lack of evidence regarding the real-world association between repurposed agents and SARS-CoV-2 infection must be addressed. Conducted under the PharmLines Initiative, this study addresses this practical problem by leveraging the unique linkage of data between the Lifelines population-based cohort and the IADB.nl pharmacy dispensing database. Our primary objective was to use a test-negative case–control (TNCC) design to rigorously evaluate the protective association between 42 candidate drugs and SARS-CoV-2 infection in a community setting. Using the TNCC framework enabled us to minimize the potential for bias in healthcare seeking that existed in previous studies [11]. Furthermore, we sought to determine whether this association is affected by dominant viral strains or individual vaccination status. By providing rigorous evidence on the possible effectiveness of these existing medications, we aim to support guidance for clinical practice and public health strategies regarding the potential usefulness of repurposing drugs for preventing COVID-19.

## 2. Results

### 2.1. Baseline Characteristics of Cases and Matched Controls

**Figure 1:**
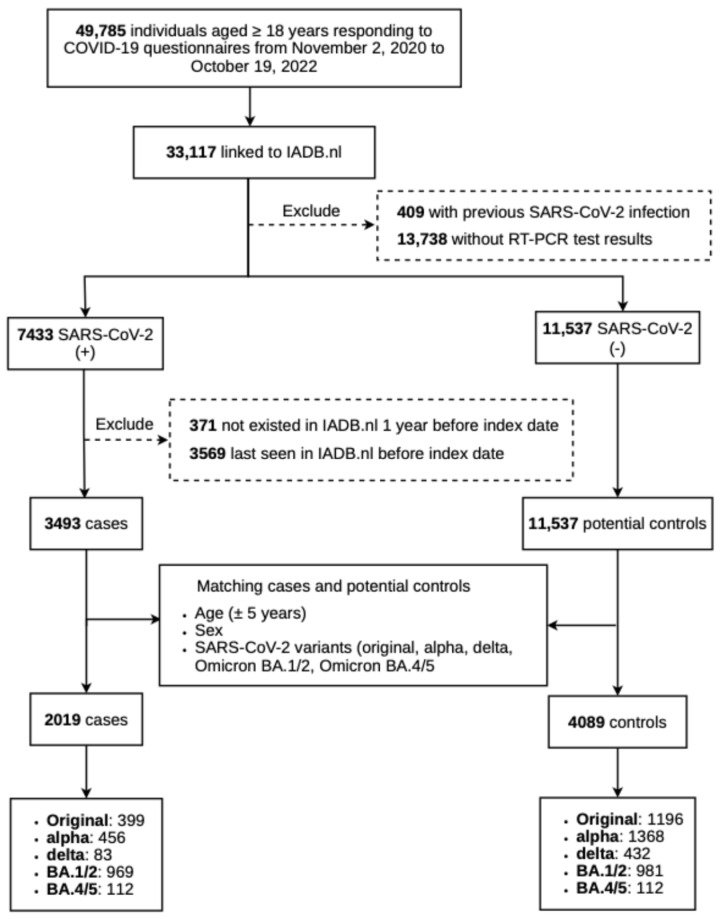
Flowchart of case and control selection and matching.

From 2 November 2020 to 19 October 2022, 49,785 adult individuals responded to the Lifelines COVID-19 questionnaire, of which 33,117 individuals were linked to IADB.nl (linkage percentage: 66.5%). After excluding individuals with previous SARS-CoV-2 infection (n = 409), individuals without RT-PCR test results (n = 13,738), individuals not present in IADB.nl one year before the index date (n = 371), and individuals last seen in IADB.nl before the index date (n = 3569), we had 3493 cases and 11,537 potential controls available for matching. After matching for age, sex, and SARS-CoV-2 variant, we included 2019 test-positive cases and 4089 matched test-negative controls in our analysis, with a case–control ratio of up to 1:3 per matched stratum (Figure 1).

Baseline characteristics of the included cases and matched controls before and after multiple imputation are shown in Table 1. Overall, the study groups were well-balanced regarding key demographic and clinical factors. Both cases and controls were predominantly female (67%) and European (over 98%), with a mean age of 56.8 years. Statistical comparisons revealed no significant differences in marital status, health cautiousness behaviors, current smoking status, BMI, or prevalence of comorbidities, indicating high comparability between the groups. However, noteworthy differences were observed in socioeconomic and behavioral variables. Compared with controls, cases had lower levels of education (18.0% low and 39.4% middle vs. 17.1% low and 36.3% middle, adjusted *p* = 0.051), were more likely to have low alcohol consumption (37.2% vs. 31.5%, adjusted *p* = 0.011), and were more likely to have been vaccinated longer ago at index date (28.1% 3–6 months and 9.7% >6 months vs. 17.5% 3–6 months and 3.0% >6 months, adjusted *p* = 0.011).

### 2.2. Associations Between Studied Drugs and Outcomes

**Figure 2:**
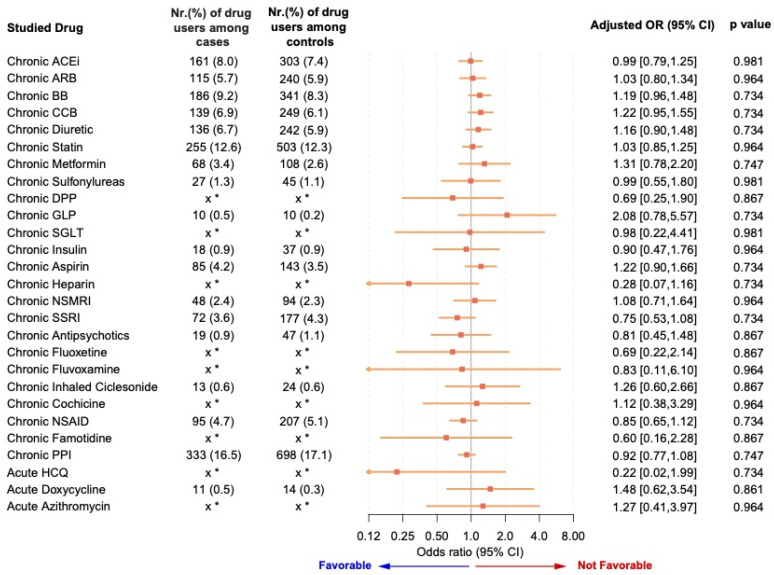
Forest plot of multivariable conditional logistic regression showing adjusted odds ratio (OR) for each candidate drug on SARS-CoV-2 infection. An asterisk (*) indicated that the number of users was smaller than 10 and not disclosable. Abbreviation: angiotensin converting enzyme inhibitors (ACEi); angiotensin receptor blockers (ARBs); beta blockers (BBs); calcium channel blockers (CCBs); dipeptidyl peptidase (DPP)-4 inhibitor; glucagon-like peptide (GLP)-1; sodium-glucose cotransporter (SGLT)-2 inhibitor; non-selective monoamine reuptake inhibitors (NSMRIs); selective serotonin reuptake inhibitor (SSRIs); nonsteroidal anti-inflammatory drugs (NSAIDs); proton-pump inhibitors (PPIs); hydroxychloroquine (HCQ).

Figure 2 summarizes the primary findings regarding the associations of the candidate drugs, and Table S1 provides full statistical details, including both crude and adjusted odds ratios. Similarly, Figure S1 visually represents the results regarding the prevention of COVID-19 hospitalization, while Table S2 provides the corresponding statistical details. After multivariable adjustment, no significant associations were observed between the studied candidate drugs and the risk of SARS-CoV-2 infection or COVID-19 hospitalization. Specific attention was paid to widely discussed repurposed agents: for example, angiotensin-converting enzyme inhibitors (ACEi) (aOR 0.99; 95% CI 0.79–1.25), angiotensin II receptor blockers (ARBs) (aOR 1.03; 95% CI 0.80–1.34), statins (aOR 1.03; 95% CI 0.85–1.25), metformin (aOR 1.31; 95% CI 0.78–2.20), and HCQ (aOR 0.22; 95% CI 0.02–1.99). Due to limited statistical power, not all studied drugs could be evaluated for their association with COVID-19 hospitalization, and only a subset is included in Figure S1. In addition, the number of ICU admissions was low and no deaths were recorded, which prevented us from performing conditional logistic regression for these outcomes.

### 2.3. Associations Stratified by SARS-CoV-2 Variants

**Figure 3:**
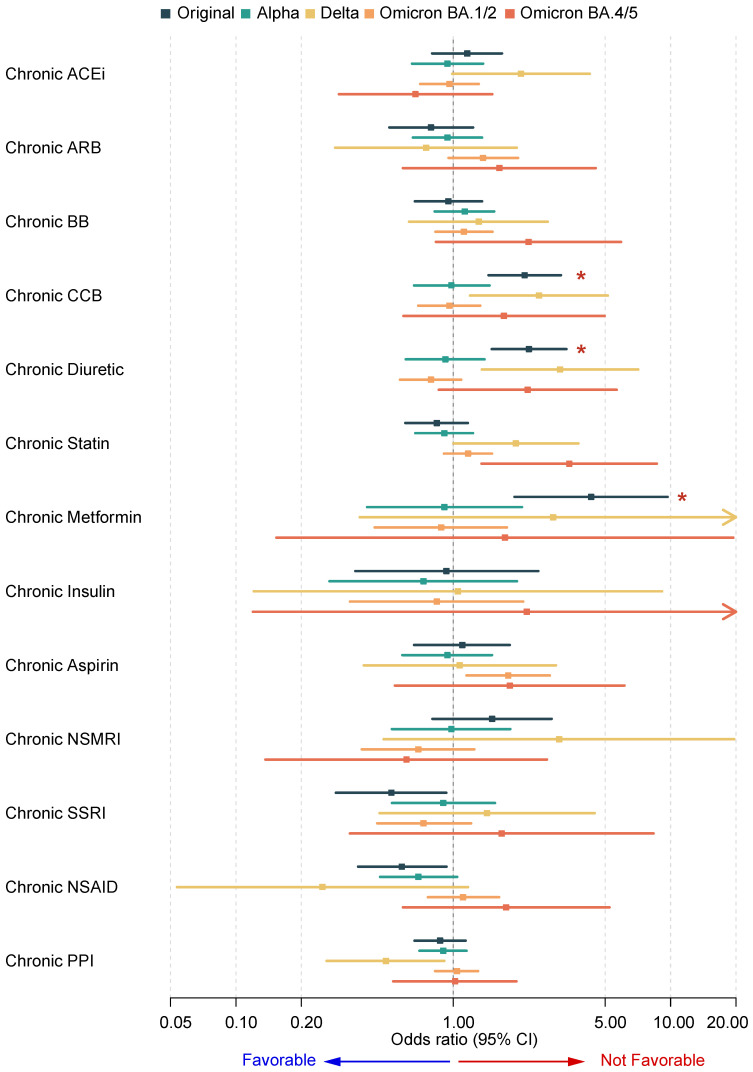
Forest plot of multivariable conditional logistic regression showing adjusted odds ratio (OR) for each candidate drug on SARS-CoV-2 infection, stratified by SARS-CoV-2 variants: Original, Alpha, Delta, Omicron BA.1/2, Omicron BA.4/5. An asterisk (*) next to the line indicates that the corrected *p*-value is >0.05. Abbreviation: angiotensin converting enzyme inhibitors (ACEi); angiotensin receptor blockers (ARBs); beta blockers (BBs); calcium channel blockers (CCBs); non-selective monoamine reuptake inhibitors (NSMRIs); selective serotonin reuptake inhibitor (SSRIs); nonsteroidal anti-inflammatory drugs (NSAIDs); proton-pump inhibitors (PPIs).

Figure 3 presents a forest plot of the association analysis, which is stratified by dominant SARS-CoV-2 variants (Original, Alpha, Delta, and Omicron subvariants). Table S3 provides the comprehensive statistical data supporting these comparisons, including both crude and multivariable-adjusted odds ratios for each variant-specific subgroup. After correcting for multiple testing, we observed only three statistically significant associations, all of which indicated an increased risk of infection with the original strain of SARS-CoV-2: chronic use of calcium channel blockers (CCBs) (aOR 2.13; 95% CI 1.45–3.13), diuretics (aOR 2.23; 95% CI 1.50–3.32), and metformin (aOR 4.31; 95% CI 1.91–9.69).

### 2.4. Associations Stratified by Vaccination Status

**Figure 4:**
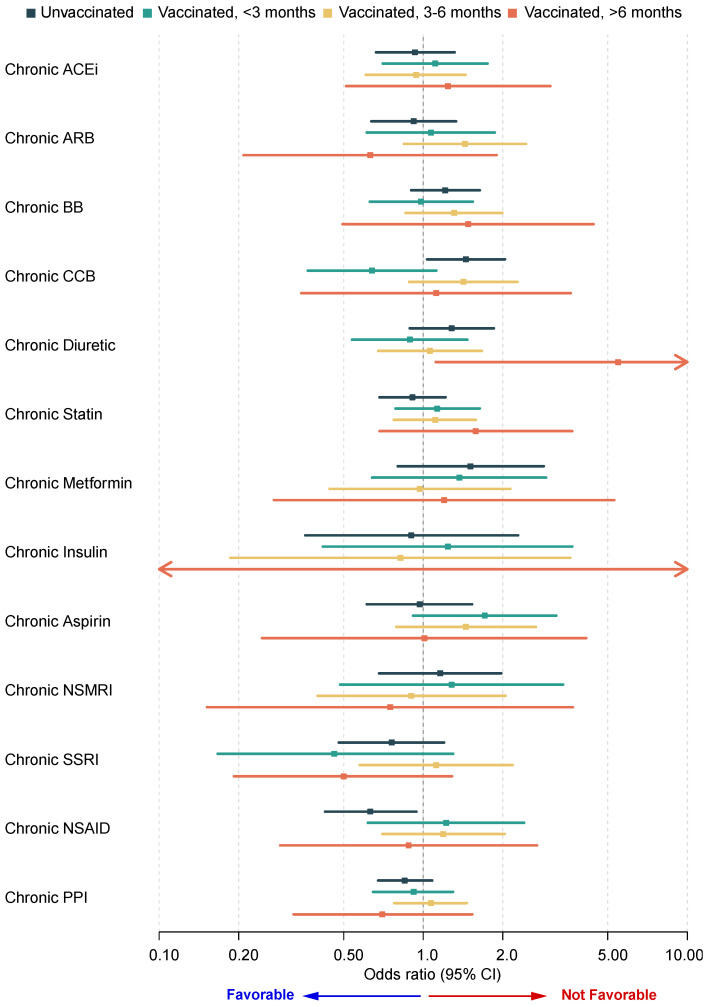
Forest plot of multivariable conditional logistic regression showing adjusted odds ratio (OR) for each candidate drug on SARS-CoV-2 infection, stratified by vaccination: unvaccinated, vaccinated <3 months, vaccinated 3–6 months, vaccinated >6 months. Abbreviation: angiotensin converting enzyme inhibitors (ACEi); angiotensin receptor blockers (ARBs); beta blockers (BBs); calcium channel blockers (CCBs); non-selective monoamine reuptake inhibitors (NSMRIs); selective serotonin reuptake inhibitors (SSRIs); nonsteroidal anti-inflammatory drugs (NSAIDs); proton-pump inhibitors (PPIs).

Figure 4 visually shows the association between each studied drug and SARS-CoV-2 infection, stratified by vaccination status. Table S4 provides the comprehensive statistical data supporting these comparisons. After correcting for multiple testing, we did not observe any statistically significant result for all studied strata. For most of the drugs, the point estimates for each vaccination status are randomly distributed around the null value (OR = 1) and no interaction term was statistically significant.

## 3. Discussion

Using the data linkage under the PharmLines initiative, our comprehensive primary analysis found no statistically significant association between the use of any of the candidate drugs for potential repurposing and the odds of SARS-CoV-2 infection. In the stratified analysis by COVID-19 variant and vaccination status, most associations remained non-statistically significant. The chronic dispensing of CCBs, diuretics, and metformin was associated with an increased odds of original strain SARS-CoV-2 infection.

Our main findings align with the results of large-scale clinical trials, such as the RECOVERY trial, as well as with our previous meta-analysis of a list of proposed candidate drugs, which demonstrated no clinical benefit for several widely discussed candidates [7,12]. Meta-analysis of case–control studies of ACEi and ARBs indicated that the pooled odds ratio for SARS-CoV-2 infection was 1.15 and 0.98 (both *p* > 0.05), respectively, while in the present TNCC study the adjusted odds ratio was 0.99 and 1.03 (both *p* > 0.05). The other negative findings of this TNCC study are consistent with previous studies by Mancia et al., Huh et al., and Reynolds et al. These studies largely found no significant association between major antihypertensive or antidiabetic drug classes and the risk of SARS-CoV-2 infection or COVID-19 severity [13,14]. These consistent findings across different populations and study designs strongly support the conclusion that these repurposed drugs do not meaningfully protect against SARS-CoV-2.

However, there are also discrepancies between our findings and some earlier published work. Mancia et al. reported a 37% increased odds of COVID-19 diagnosis among active insulin users [15], a finding not replicated in our study. In addition, while they did not observe a significant association with CCBs (aOR = 1.03; 95% CI 0.95–1.12), diuretics (aOR = 1.03; 95% CI 0.86–1.23), and oral diabetes drugs (aOR = 1.07; 95% CI 0.97–1.17) during the original strain [15], our study found that CCBs, diuretics, and metformin use were associated with an increased risk of SARS-CoV-2 infection with the original strain. Similarly, a cohort study conducted during the same period by Reynolds et al. reported a 6% lower odds of testing positive for COVID-19 among BB users [14], but we did not observe this protective association of BB use. The protective association of statins reported by Fung et al. [16] was not confirmed in our study either. We argue that these discrepancies likely stem from methodological differences rather than true biological variation. In our study, we used a more stringent exposure definition, requiring at least two dispensings within 270 days, to ensure active drug use. Previous studies used broader windows of up to 18 months, which may not accurately reflect current medication use. In addition, we addressed healthcare-seeking bias, which may have distorted earlier results. Consequently, our study provides a more refined evaluation that calls into question the marginal protective or harmful effects reported in less strictly controlled observational cohorts.

The impact of antihypertensives and antidiabetics on susceptibility to SARS-CoV-2 infection is still being debated. Interpreting these relationships requires careful consideration of biological and methodological factors. For example, diagnosed hypertension has been associated with a slightly increased risk of COVID-19 mortality (hazard ratio 1.07; 95% CI 1.00–1.15), though this association is likely confounded by age and comorbidities such as diabetes and obesity [17]. Patients with diabetes are at an increased risk for lower respiratory tract infections [18], and improved glycemic control through medications could reduce viral replication and immune dysregulation [19]. However, residual confounding factors from the severity of the underlying disease and disease–drug interactions cannot be entirely ruled out in real-world studies.

It has been speculated that the association between studied drugs and outcomes may vary as SARS-CoV-2 evolves, particularly due to shifts in viral entry pathways. While earlier variants relied heavily on transmembrane protease serine 2 (TMPRSS2)-dependent entry at the cell surface, the Omicron variant shifted toward a more prominent endosomal entry pathway [20]. In theory, this mechanistic transition could alter the efficacy of drugs that target specific entry points. However, in our subgroup analysis focused on the Omicron period, no significant association was found between studied drugs and SARS-CoV-2 infection. This suggests that, regardless of shifts in viral entry mechanisms or specific clinical phases of the pandemic, these repurposed agents did not provide detectable protection in real-world settings.

Similarly, for the studied drugs with antiviral and immunomodulatory effects such as immune cell adhesion and migration [21], it is plausible that vaccine-induced immunity could interact with their mechanisms of action. For example, Paxlovid was shown to reduce the risk of COVID-19 hospitalization by approximately 89% in unvaccinated high-risk patients [22], but only 51% in those who had received vaccine booster doses [23]. Pidotimod has also demonstrated potential in reducing disease severity and the risk of hospitalization, particularly among unvaccinated populations [24,25]. However, our TNCC study did not observe any evidence supporting such a hypothesis.

One of the main advantages of our study is the use of a TNCC design, which helps to minimize temporal variation in health-seeking behavior. By matching cases and controls based on the questionnaire response date, we ensured that observed differences in association are primarily attributed to the studied drug rather than fluctuations in health-seeking behavior over time. Additionally, this approach reduces temporal variation in the likelihood of SARS-CoV-2 infection, further enhancing the robustness of our findings. Moreover, we considered the effect modification of COVID-19 vaccination and evaluated the association against different SARS-CoV-2 variants of the studied drugs, providing a comprehensive assessment of its impact and thus guiding future studies.

Our observational study has limitations. First, we prioritized outcome accuracy by including only self-reported RT-PCR test results from the GGD, because self-tests are known to be less sensitive [26]. While this approach minimizes outcome misclassification, it introduces a trade-off regarding selection bias. By excluding individuals who relied solely on home-based self-tests, who may have had milder symptoms or different healthcare-seeking behaviors, our final analytic sample may not fully represent the entire spectrum of SARS-CoV-2 infections in the community. Nevertheless, we believe the diagnostic specificity gained from RT-PCR results outweighs the potential risk of selection bias, especially when evaluating the subtle associations of repurposed medications. Second, due to the nature of a questionnaire-based study, where only non-severely ill individuals are able to respond, our study population primarily consists of relatively healthy individuals. However, the purpose of our study was to evaluate the value of the studied drugs for the community setting, rather than for a high-risk population. As a result, there were few hospitalized cases and virtually no cases of ICU admission or deaths. Third, we only considered the first SARS-CoV-2 infection to exclude the effect of natural immunity. However, missing data on previous infections remains a concern because some participants with previous infections may have been enrolled without reporting their infection history, though this is highly unlikely given the repeated digital measurements taken during the pandemic. Fourth, after 11 April 2022, confirmatory testing at the GGD was no longer required for positive self-tests, which may have led to underreporting of COVID-19 cases. Although the test-negative case–control design helps to mitigate temporal variation in testing probability through time matching, shifts in testing behavior could still introduce bias. More severe cases may have been tested at the GGD later in the study period, while less severe cases may also have sought testing due to factors such as travel mandates. Fifth, some variables, such as household composition and income, were not considered in the analyses due to excessive missing data, which may have affected our ability to fully adjust for socio-economic differences. However, as shown in Table 1, differences in measured characteristics were not substantial due to the TNCC design.

Lastly, we conducted post hoc power simulations using the actual number of cases and controls included in our study (see Figure S2). The results indicated that, although the study was robustly powered (>95%) to detect strong protective associations (OR ≤ 0.5) at low exposure prevalence, sensitivity decreased for more moderate associations. Specifically, the power to detect an OR of 0.7 was approximately 41% for medications with an exposure prevalence of 2%. This suggests a potential risk of type II errors for less frequently used drugs, where modest clinical benefits might remain undetected. Therefore, future studies examining these drugs should aim for a significantly larger sample size to achieve adequate statistical power.

These results ultimately highlight the practical challenges of drug repurposing and provide a rigorous, real-world evaluation of existing clinical hypotheses. Importantly, in this observational framework, any observed associations, particularly those suggesting an increased risk in specific subgroups, should be interpreted as correlations, not causal links. Understanding this broader context is essential for interpreting the association between current prophylactic strategies and SARS-CoV-2. In conclusion, despite limited statistical power for some drugs, none of the studied drugs showed protective associations against SARS-CoV-2 infection. These findings do not support the off-label use of these agents for prophylaxis in the general population. To build upon these results, future research should prioritize large-scale, multicenter collaborative studies to achieve the statistical power necessary to evaluate drugs with low prescribing prevalence. Additionally, prospective studies that incorporate real-time viral sequencing would enable a more precise evaluation of the association between these drugs and specific emerging subvariants. Finally, investigating the impact of drug dosage and duration of use is critical for clarifying the potential prophylactic role of these agents.

## 4. Materials & Methods

### 4.1. Study Design, Setting and Data Sources

We conducted a TNCC study to evaluate the association between the studied drug use and the risk of SARS-CoV-2 infection and severe COVID-19. We conducted this study under the PharmLines Initiative, which linked the data of the Lifelines (https://www.lifelines.nl/) and the University of Groningen community pharmacy dispensing database IADB.nl (https://iadb.nl/). Lifelines is a multi-disciplinary prospective population-based cohort study examining in a unique three-generation design the health and health-related behaviours of 167,729 persons living in the North of the Netherlands. It employs a broad range of investigative procedures in assessing the biomedical, socio-demographic, behavioural, physical and psychological factors which contribute to the health and disease of the general population, with a special focus on multi-morbidity and complex genetics [27,28].

The Lifelines COVID-19 cohort is a prospective and population-based cohort study examining the health and health-related behaviors specifically related to COVID-19 within the Lifelines cohort which started during the pandemic [29]. The digital questionnaires were sent out starting March 2020 and continued through late 2022. In the current study, data from 16 consecutive questionnaires were used, spanning from 2 November 2020 till 19 October 2022.

IADB.nl is an evolving pharmacy drug dispensing database that contains prescription data for more than 1.2 million participants from approximately 120 community pharmacies. The vaccination data were retrieved from the COVID-19-vaccination Information and Monitoring System (CIMS) on 1 May 2024. CIMS is a national vaccination registration system maintained by the Dutch National Institute for Public Health and the Environment (RIVM).

A Trusted Third Party, Statistics Netherlands (Dutch: Centraal Bureau voor de Statistiek, CBS), carried out the linkage of the Lifelines and the IADB.nl records at the patient level based on postal code, sex and date of birth [30].

### 4.2. Study Population

We included individuals aged ≥ 18 years who reported at least one reverse transcription polymerase chain reaction (RT-PCR) test result from COVID-19 questionnaire 15b (2 November 2020) onward. As of assessment 15b onwards, the question “were the tests performed by GGD” was included in the questionnaire due to the increased availability of self-administered rapid antigen tests since November 2020. We restricted the inclusion of population based on data from RT-PCR-tests performed by the Dutch municipal health service (Gemeentelijke gezondheidsdienst, GGD) during the pandemic. We excluded information from self-tests or other laboratory tests to ensure accuracy. All cases and controls were registered in the IADB.nl database to retrieve information on drug dispensing. We excluded individuals who ever reported a previous SARS-CoV-2 infection before the start of the study period. A previous infection was defined as a positive test by PCR at the GGD at any time point between 30 March 2020 (COVQ 1) and 1 November 2020 (COVQ 15).

Cases were defined as individuals with a first-time self-reported positive RT-PCR test for SARS-CoV-2 during the study period. Re-infections were excluded. The index date for each case was the questionnaire response date of reporting the first SARS-CoV-2 test.

Controls were those who reported only negative RT-PCR tests throughout the study period. The definition of cases and controls was consistent with that previously stated in other published test-negative case–control studies [31,32]. Controls were matched to cases by age (±5 years), sex, and calendar period in which the RT-PCR test was taken. The calendar period served as a proxy for the most dominant SARS-CoV-2 variant that circulated during that period (Table 2). The controls were assigned the same index date as their corresponding cases. If an individual had multiple negative tests, we selected the one to be matched to obtain the highest possible case-to-control ratio. Both cases and controls should be in the IADB.nl one year before the index date, and the last date recorded in the IADB.nl should be after the index date to ensure follow-up.

### 4.3. Exposure to Studied Drugs Repurposing for COVID-19

The selection of the drugs studied in our study for prophylactic use against SARS-CoV-2 was based on our previous systematic review, which summarized the existing drugs that could potentially be repurposed for COVID-19 [7]. We also conducted additional searches for any published reports on new repurposed drugs beyond those reported in our systematic review. The studied drugs of interest and corresponding Anatomical Therapeutic Chemical (ATC) code are summarized in Table S5.

Chronic drug use was defined as at least two dispensings recorded in the IADB.nl database within a period of 1 to 270 days before the index date for each individual. For drugs dispensed for only a short time (an acute drug), this was defined as at least one dispensing 1–30 days before the index date. Otherwise, drug exposure was defined as absent.

### 4.4. Outcomes

The primary outcome was any SARS-CoV-2 infection as determined by a self-reported positive RT-PCR test performed at the GGD. The questions used to define outcomes in the Lifelines COVID-19 cohort are listed in Table S6. Severe COVID-19 was the exploratory outcome and was recorded as follows: hospitalization after SARS-CoV-2 infection, ICU admission after SARS-CoV-2 infection, or death. Only mortality within 3 months after the index date of the SARS-CoV-2 infection in cases was considered.

### 4.5. Covariates

The selection of covariates was based on prior research and available variables from the Lifelines cohort, Lifelines COVID-19 questionnaires, CIMS data, or IADB.nl dispensing data. We considered the following covariates as potential risk factors for the case status: age at index date, sex, ethnicity, educational level, marital status, vaccination status at index date, health cautiousness behavior, current smoking, body mass index (BMI), alcohol consumption, and comorbidity at index date. Comorbidities were classified as cardiovascular disease (CVD), lung disease, diabetes, psychological, cancer, and neurological. The full details on data retrieval and definitions of covariates are summarized in Table S7. For some covariates with repeated assessments at different time points, we chose the assessment closest to the index date because it is the most temporally relevant.

### 4.6. Statistical Analysis

The baseline characteristics of the variables are summarized using means and standard deviations (SDs) for continuous variables and frequencies and percentages for categorical variables. We compared the differences in continuous variables between cases and controls using the *t*-test, while the differences in categorical variables were tested using the chi-square test or Fisher’s exact test.

Missing covariate data (ethnicity, education, marital status, health cautiousness behavior, smoking, BMI, and alcohol consumption) was assumed to be missing at random and was handled via multiple imputation via the MICE package using 20 imputations [33]. Each studied drug was analyzed with a separate imputation model. We estimated the association between exposure and outcomes using multivariable conditional logistic regression with adjustment for confounders. We fitted a separate regression model for each studied drug to examine its association with the outcome. Each case and its matched controls were considered a single group within the regression model. The adjusted variables in the model included age, sex, marital status, educational level, SARS-CoV-2 variant, vaccination status, comorbidities, health cautiousness behavior, ethnicity, and alcohol consumption. The results of the logistic regression analyses are presented as odds ratios (ORs) with 95% confidence intervals (CIs). Forest plots were used to visualize the results.

We also performed stratified analyses to examine potential effect modification of SARS-CoV-2 variant and COVID-19 vaccine. First, we stratified analyses by the SARS-CoV-2 variants the case individuals were infected with (original, Alpha, Delta, Omicron BA.1/BA.2, Omicron BA.4/BA.5). As shown in Table 2, these variant classifications were defined by specific calendar periods based on dominant viral circulation. This temporal framework served as the basis for the variant-stratified results presented in the subsequent sections. Second, we considered potential effect modification by COVID-19 vaccination by including the interaction term of studied drug use and vaccination status (unvaccinated, vaccinated <3 months, vaccinated 3–6 months, vaccinated >6 months).

All statistical analyses were performed using R (version 4.4.0). We used the Benjamini–Hochberg approach to adjust for multiple comparisons and reduce the number of false positives. The significance threshold for all statistical tests was a two-sided *p*-value < 0.05.

## Figures and Tables

**Table 1:** Baseline characteristics of cases (N = 2019) and matched controls (N = 4089) before and after multiple imputation.

	Before Multiple Imputation				After Multiple Imputation			
Variable	Case	Control	SMD	*p* Value ^¶^	Case	Control	SMD	*p* Value ^¶^
Number	2019	4089			2019	4089		
Age (mean, SD)	56.84 (12.27)	56.84 (12.31)	<0.001	1.000	56.84 (12.27)	56.84 (12.31)	<0.001	1.000
BMI (mean, SD)	26.58 (4.50)	26.39 (4.46)	0.040	0.453	26.58 (4.50)	26.39 (4.46)	0.040	0.453
Gender (female)	1348 (66.8)	2747 (67.2)	0.009	0.939	1348 (66.8)	2747 (67.2)	0.009	0.939
Ethnicity			0.091	0.189			0.088	0.202
White/eastern and western European	1874 (98.7)	3725 (98.2)			1994 (98.8)	4021 (98.3)		
Other ^‡^	25 (1.3)	68 (1.8)			25 (1.2)	68 (1.7)		
Missing	120	296						
Educational level ^†^			0.095	0.081			0.098	0.051
Low	X	697 (17.6)			X	699 (17.1)		
Middle	779 (39.5)	1447 (36.6)			796 (39.4)	1483 (36.3)		
High	827 (41.9)	1797 (45.4)			859 (42.5)	1891 (46.2)		
Other	X	16 (0.4)			X	16 (0.4)		
Missing	47	132						
Marital status			0.102	0.066			0.086	0.116
Single	142 (7.7)	362 (10.2)			144 (7.1)	380 (9.3)		
Registered partnership	205 (11.1)	432 (12.2)			218 (10.8)	476 (11.6)		
Married	1239 (67.1)	2241 (63.3)			1397 (69.2)	2729 (66.7)		
Other	260 (14.1)	504 (14.2)			260 (12.9)	504 (12.3)		
Missing	173	550						
Cautiousness								
Not cautious	513 (30.8)	1072 (32.8)	0.043	0.462	527 (26.1)	1129 (27.6)	0.034	0.614
Missing	355	822						
Current smoking (no)	1744 (91.2)	3444 (90.2)	0.036	0.552	1851 (91.7)	3714 (90.8)	0.030	0.647
Missing	107	270						
BMI			0.040	0.814			0.042	0.814
Underweight	10 (0.5)	25 (0.6)			10 (0.5)	25 (0.6)		
Normal	798 (41.0)	1605 (41.1)			846 (41.9)	1710 (41.8)		
Overweight	783 (40.2)	1593 (40.8)			803 (39.8)	1667 (40.8)		
Obese	358 (18.4)	680 (17.4)			360 (17.8)	687 (16.8)		
Missing	70	186						
Alcohol consumption			0.074	0.189			0.121	0.011
Low	703 (38.5)	1125 (35.1)			752 (37.2)	1289 (31.5)		
Moderate	1093 (59.9)	2014 (62.9)			1239 (61.4)	2738 (67.0)		
High	28 (1.5)	62 (1.9)			28 (1.4)	62 (1.5)		
Missing	195	888						
Vaccination status			0.417	0.022			0.421	0.011
Unvaccinated	889 (44.2)	2440 (60.1)			892 (44.2)	2464 (60.3)		
Vaccinated, <3 months	365 (18.1)	784 (19.3)			365 (18.1)	786 (19.2)		
Vaccinated, 3–6 months	563 (28.0)	716 (17.6)			567 (28.1)	717 (17.5)		
Vaccinated, >6 months	195 (9.7)	122 (3.0)			195 (9.7)	122 (3.0)		
Missing	7	27						
CVD	944 (46.8)	1913 (46.8)	0.001	1.000	944 (46.8)	1913 (46.8)	0.001	1.000
Hypertension	594 (29.4)	1108 (27.1)	0.052	0.224	594 (29.4)	1108 (27.1)	0.052	0.224
High cholesterol	547 (27.1)	1137 (27.8)	0.016	0.814	547 (27.1)	1137 (27.8)	0.016	0.814
Diabetes	115 (5.7)	203 (5.0)	0.033	0.552	115 (5.7)	203 (5.0)	0.033	0.552
Lung disease	457 (22.6)	914 (22.4)	0.007	0.960	457 (22.6)	914 (22.4)	0.007	0.960
Asthma/COPD	237 (11.7)	499 (12.2)	0.014	0.814	237 (11.7)	499 (12.2)	0.014	0.814
Cancer	92 (4.6)	204 (5.0)	0.020	0.814	92 (4.6)	204 (5.0)	0.020	0.814
Psychological	259 (12.8)	560 (13.7)	0.026	0.740	259 (12.8)	560 (13.7)	0.026	0.740
Depression	216 (10.7)	460 (11.2)	0.018	0.814	216 (10.7)	460 (11.2)	0.018	0.814
Neurological	13 (0.6)	33 (0.8)	0.019	0.814	13 (0.6)	33 (0.8)	0.019	0.814

^¶^ Corrected for multiple testing using Benjamini–Hochberg approach. ^‡^ The “other” category of ethnicity included White/Mediterranean or Arabic, Asian, Black/Negroid, and the other minor ethnicities. ^†^ There are some individuals who reported educational level that cannot be categorized as low, middle, or high, and therefore categorized as “other”. However, due to the small number of individuals in this category and the risk of disclosure, the number of individuals with “other” educational levels is not shown and replaced with X in the table. Abbreviation: standardized mean difference (SMD); standard deviation (SD); body mass index (BMI); cardiovascular disease (CVD); chronic obstructive pulmonary disease (COPD).

**Table 2:** SARS-CoV-2 variants and their corresponding calendar period.

SARS-CoV-2 Variant	Calendar Period ^†^
Original	Positive test date between 27 February 2020 and 31 January 2021 (more than 72% cases were original strain infections)
Alpha	Positive test date between 22 February 2021 and 13 June 2021 (more than 75% cases were alpha infections)
Delta	Positive test date between 5 July 2021 and 19 December 2021 (more than 80% cases were delta infections)
Omicron BA.1/BA.2	Positive test date between 3 January 2022 and 6 June 2022 (more than 85% cases were BA.1 or BA.2 infections)
Omicron BA.4/BA.5	Positive test date between 20 June 2022 and 19 October 2022 (more than 85% cases were BA.4 or BA.5 infections)

^†^ The calendar period definition is based on information from the Rijksinstituut voor Volksgezondheid en Milieu (RIVM). Varianten van het coronavirus SARS-CoV-2 (2022). https://www.rivm.nl/coronavirus-covid-19/virus/varianten (accessed on 1 May 2024).

## Data Availability

Data supporting the findings are available as part of the manuscript or from the Supplementary Files. The corresponding author may grant access to the dataset upon reasonable request, subject to approval from the relevant data providers and ethical committees. The R code for data analysis can be shared by emailing the corresponding author.

## Supplementary Materials

The following supporting information can be downloaded at: https://www.mdpi.com/article/10.3390/ph19060861/s1. **Table S1**: Conditional logistic regression analysis – crude and adjusted odds ratios (OR) and 95% confidence interval (lower and upper limit) of candidate drugs for repurposing for prevention of SARS-CoV-2 infection; **Table S2**: Conditional logistic regression analysis – crude and adjusted odds ratios (OR) and 95% confidence interval (lower and upper limit) of candidate drugs for repurposing for prevention of COVID-19 hospitalization; **Figure S1**: Forest plot of multivariable conditional logistic regression showing adjusted odds ratio (OR) for each candidate drug for the prevention of COVID-19 hospitalization; **Table S3**: Conditional logistic regression analysis – crude and adjusted odds ratios (OR) and 95% confidence interval of candidate drugs for SARS-CoV-2 infection, stratified by SARS-CoV-2 variants; **Table S4**: Conditional logistic regression analysis – crude and adjusted odds ratios (OR) and 95% confidence interval of candidate drugs for SARS-CoV-2 infection, stratified by vaccination status; **Figure S2**: Heat plot illustrating the study power at different levels of odds ratios and exposure prevalence; **Table S5**: List of candidate drugs of interest and corresponding ATC code; **Table S6**: Outcome definitions based on the Lifelines COVID-19 cohort questions; **Table S7**: Definition of covariates.

## References

[1] Parker E.P.K., Desai S., Marti M., Nohynek H., Kaslow D.C., Kochhar S., O’Brien K.L., Hombach J., Wilder-Smith A. (2022). Response to Additional COVID-19 Vaccine Doses in People Who Are Immunocompromised: A Rapid Review. Lancet Glob. Health.

[2] Our World in Data Global Data on COVID-19 Vaccinations. https://ourworldindata.org/covid-vaccinations.

[3] Food and Drug Administration (FDA) Coronavirus (COVID-19) Update: FDA Authorizes New Long-Acting Monoclonal Antibodies for Pre-Exposure Prevention of COVID-19 in Certain Individuals. https://www.fda.gov/news-events/press-announcements/coronavirus-covid-19-update-fda-authorizes-new-long-acting-monoclonal-antibodies-pre-exposure.

[4] Food and Drug Administration (FDA) FDA Announces Evusheld Is Not Currently Authorized for Emergency Use in the U.S. https://www.astrazeneca.com/media-centre/press-releases/2023/update-on-evusheld-us-eua.html.

[5] Patel P., Wentworth D.E., Daskalakis D. (2024). COVID-19 Therapeutics for Nonhospitalized Older Adults. JAMA.

[6] Venkatesan P. (2021). Repurposing Drugs for Treatment of COVID-19. Lancet Respir. Med..

[7] Zhou G., Verweij S., Bijlsma M.J., de Vos S., Oude Rengerink K., Pasmooij A.M.G., van Baarle D., Niesters H.G.M., Mol P., Vonk J.M. (2023). Repurposed Drug Studies on the Primary Prevention of SARS-CoV-2 Infection during the Pandemic: Systematic Review and Meta-Analysis. BMJ Open Respir. Res..

[8] WHO Living Guideline: Drugs to Prevent COVID-19. https://www.who.int/publications/i/item/WHO-2019-nCoV-prophylaxes-2023.1.

[9] Lamontagne F., Agoritsas T., Siemieniuk R., Rochwerg B., Bartoszko J., Askie L., MacDonald H., Amin W., Bausch F.J., Burhan E. (2021). A Living WHO Guideline on Drugs to Prevent Covid-19. BMJ.

[10] European Medicines Agency EMA Advises Against Use of Ivermectin for the Prevention or Treatment of COVID-19 Outside Randomised Clinical Trials. https://www.ema.europa.eu/en/news/ema-advises-against-use-ivermectin-prevention-treatment-covid-19-outside-randomised-clinical-trials.

[11] Lipsitch M., Tchetgen Tchetgen E., Cohen T. (2010). Negative Controls: A Tool for Detecting Confounding and Bias in Observational Studies. Epidemiology.

[12] The RECOVERY Collaborative Group (2020). Effect of Hydroxychloroquine in Hospitalized Patients with Covid-19. N. Engl. J. Med..

[13] Huh K., Ji W., Kang M., Hong J., Bae G.H., Lee R., Na Y., Jung J. (2021). Association of Prescribed Medications with the Risk of COVID-19 Infection and Severity among Adults in South Korea. Int. J. Infect. Dis..

[14] Reynolds H.R., Adhikari S., Pulgarin C., Troxel A.B., Iturrate E., Johnson S.B., Hausvater A., Newman J.D., Berger J.S., Bangalore S. (2020). Renin–Angiotensin–Aldosterone System Inhibitors and Risk of Covid-19. N. Engl. J. Med..

[15] Mancia G., Rea F., Ludergnani M., Apolone G., Corrao G. (2020). Renin–Angiotensin–Aldosterone System Blockers and the Risk of Covid-19. N. Engl. J. Med..

[16] Fung K.W., Baik S.H., Baye F., Zheng Z., Huser V., McDonald C.J. (2022). Effect of Common Maintenance Drugs on the Risk and Severity of COVID-19 in Elderly Patients. PLoS ONE.

[17] Williamson E.J., Walker A.J., Bhaskaran K., Bacon S., Bates C., Morton C.E., Curtis H.J., Mehrkar A., Evans D., Inglesby P. (2020). OpenSAFELY: Factors Associated with COVID-19 Death in 17 Million Patients. Nature.

[18] Muller L.M.A.J., Gorter K.J., Hak E., Goudzwaard W.L., Schellevis F.G., Hoepelman A.I.M., Rutten G.E.H.M. (2005). Increased Risk of Common Infections in Patients with Type 1 and Type 2 Diabetes Mellitus. Clin. Infect. Dis..

[19] Liu J.-W., Huang X., Wang M.-K., Yang J.-S. (2024). Diabetes and Susceptibility to COVID-19: Risk Factors and Preventive and Therapeutic Strategies. World J. Diabetes.

[20] Willett B.J., Grove J., MacLean O.A., Wilkie C., De Lorenzo G., Furnon W., Cantoni D., Scott S., Logan N., Ashraf S. (2022). SARS-CoV-2 Omicron Is an Immune Escape Variant with an Altered Cell Entry Pathway. Nat. Microbiol..

[21] Zeiser R. (2018). Immune Modulatory Effects of Statins. Immunology.

[22] Hammond J., Leister-Tebbe H., Gardner A., Abreu P., Bao W., Wisemandle W., Baniecki M., Hendrick V.M., Damle B., Simón-Campos A. (2022). Oral Nirmatrelvir for High-Risk, Nonhospitalized Adults with Covid-19. N. Engl. J. Med..

[23] Shah M.M., Joyce B., Plumb I.D., Sahakian S., Feldstein L.R., Barkley E., Paccione M., Deckert J., Sandmann D., Gerhart J.L. (2022). Paxlovid Associated with Decreased Hospitalization Rate Among Adults with COVID-19—United States, April–September 2022. MMWR. Morb. Mortal. Wkly. Rep..

[24] Ucciferri C., Chiappini F., Vecchiet J., Falasca K. (2025). From Legacy to Innovation: Pidotimod’s Expanding Therapeutic Horizon. Mediterr. J. Hematol. Infect. Dis..

[25] Ucciferri C., Di Gasbarro A., Borrelli P., Di Nicola M., Vecchiet J., Falasca K. (2022). New Therapeutic Options in Mild Moderate COVID-19 Outpatients. Microorganisms.

[26] Venekamp R.P., Schuit E., Hooft L., Veldhuijzen I.K., van den Bijllaardt W., Pas S.D., Zwart V.F., Lodder E.B., Hellwich M., Koppelman M. (2023). Diagnostic Accuracy of SARS-CoV-2 Rapid Antigen Self-Tests in Asymptomatic Individuals in the Omicron Period: A Cross-Sectional Study. Clin. Microbiol. Infect..

[27] Stolk R.P., Rosmalen J.G.M., Postma D.S., De Boer R.A., Navis G., Slaets J.P.J., Ormel J., Wolffenbuttel B.H.R. (2008). Universal Risk Factors for Multifactorial Diseases: LifeLines: A Three-Generation Population-Based Study. Eur. J. Epidemiol..

[28] Scholtens S., Smidt N., Swertz M.A., Bakker S.J.L., Dotinga A., Vonk J.M., Van Dijk F., Van Zon S.K.R., Wijmenga C., Wolffenbuttel B.H.R. (2015). Cohort Profile: LifeLines, a Three-Generation Cohort Study and Biobank. Int. J. Epidemiol..

[29] McIntyre K., Lanting P., Deelen P., Wiersma H.H., Vonk J.M., Ori A.P.S., Jankipersadsing S.A., Warmerdam R., Van Blokland I., Boulogne F. (2021). Lifelines COVID-19 Cohort: Investigating COVID-19 Infection and Its Health and Societal Impacts in a Dutch Population-Based Cohort. BMJ Open.

[30] Sediq R., van der Schans J., Dotinga A., Alingh R.A., Wilffert B., Bos J.H.J., Schuiling-Veninga C.C.M., Hak E. (2018). Concordance Assessment of Self-Reported Medication Use in the Netherlands Three-Generation Lifelines Cohort Study with the Pharmacy Database Iadb.Nl: The Pharmlines Initiative. Clin. Epidemiol..

[31] Tartof S.Y., Slezak J.M., Puzniak L., Hong V., Frankland T.B., Ackerson B.K., Xie F., Takhar H., Ogun O.A., Simmons S. (2023). Effectiveness of BNT162b2 BA.4/5 Bivalent MRNA Vaccine against a Range of COVID-19 Outcomes in a Large Health System in the USA: A Test-Negative Case–Control Study. Lancet Respir. Med..

[32] Kirsebom F.C.M., Andrews N., Stowe J., Ramsay M., Lopez Bernal J. (2023). Duration of Protection of Ancestral-Strain Monovalent Vaccines and Effectiveness of Bivalent BA.1 Boosters against COVID-19 Hospitalisation in England: A Test-Negative Case-Control Study. Lancet Infect. Dis..

[33] van Buuren S., Groothuis-Oudshoorn K. (2011). Mice: Multivariate Imputation by Chained Equations in R. J. Stat. Softw..

