# Supplementary material for: Repurposing Candidate Drugs to Prevent SARS-CoV-2: A PharmLines Test-Negative Case–Control Study
Source: Pharmaceuticals (Basel). 2026 May 29;19(6):861. doi: 10.3390/ph19060861 (PMC13304682; doi:10.3390/ph19060861)
Supplement: Supplementary file 1 [file pharmaceuticals-19-00861-s001.zip › pharmaceuticals-4256886-supplementary.pdf]

Supplementary information for Real-World Study on Candidate Drugs Proposed for Repurposing to Prevent SARS-CoV-2 Infection: Test-Negative Case-Control Study under the PharmLines Initiative

Table of Contents

Table S1 Conditional logistic regression analysis – crude and adjusted odds ratios (OR) and 95% confidence interval (lower and upper limit) of candidate drugs for repurposing for prevention of SARS-CoV-2 infection 3

Table S2 Conditional logistic regression analysis – crude and adjusted odds ratios (OR) and 95% confidence interval (lower and upper limit) of candidate drugs for repurposing for prevention of COVID-19 hospitalization 5

Figure S1 Forest plot of multivariable conditional logistic regression showing adjusted odds ratio (OR) for each candidate drug for the prevention of COVID-19 hospitalization 6

Table S3 Conditional logistic regression analysis – crude and adjusted odds ratios (OR) and 95% confidence interval of candidate drugs for SARS-CoV-2 infection, stratified by SARS-CoV-2 variants 7

Table S4 Conditional logistic regression analysis – crude and adjusted odds ratios (OR) and 95% confidence interval of candidate drugs for SARS-CoV-2 infection, stratified by vaccination status 10

Figure S2 Heat plot illustrating the study power at different levels of odds ratios and exposure prevalence 12

Table S5 List of candidate drugs of interest and corresponding ATC code 13

Table S6 Outcome definitions based on the Lifelines COVID-19 cohort questions 15

Table S7 Definition of covariates 16

Lifelines Corona Research Initiative 19

Table S1 Conditional logistic regression analysis – crude and adjusted odds ratios (OR) and 95% confidence interval (lower and upper limit) of candidate drugs for repurposing for prevention of SARS-CoV-2 infection

Candidate drug	Crude OR	p value	Lower limit	Upper limit	Adjusted OR	Lower limit	Upper limit	p value ¶
Chronic ACEi	0.99	0.894	0.80	1.22	0.99	0.79	1.25	0.981
Chronic ARB	0.98	0.878	0.77	1.25	1.03	0.80	1.34	0.964
Chronic BB	1.09	0.416	0.89	1.33	1.19	0.96	1.48	0.734
Chronic CCB	1.08	0.502	0.86	1.36	1.22	0.95	1.55	0.734
Chronic diuretics	1.03	0.768	0.82	1.30	1.16	0.90	1.48	0.734
Chronic statin	0.93	0.435	0.78	1.11	1.03	0.85	1.25	0.964
Chronic metformin	1.17	0.337	0.85	1.62	1.31	0.78	2.20	0.747
Chronic sulfonylureas	1.02	0.930	0.62	1.68	0.99	0.55	1.80	0.981
Chronic DPP	0.74	0.520	0.29	1.86	0.69	0.25	1.90	0.867
Chronic GLP	1.88	0.176	0.75	4.68	2.08	0.78	5.57	0.734
Chronic SGLT	1.04	0.954	0.27	4.05	0.98	0.22	4.41	0.981
Chronic insulin	0.99	0.964	0.55	1.77	0.90	0.47	1.76	0.964
Chronic aspirin	1.18	0.264	0.88	1.58	1.22	0.90	1.66	0.734
Chronic heparin	0.45	0.227	0.13	1.64	0.28	0.07	1.16	0.734
Chronic NSMRI	1.02	0.924	0.71	1.47	1.08	0.71	1.64	0.964
Chronic SSRI	0.83	0.219	0.62	1.11	0.75	0.53	1.08	0.734
Chronic antipsychotics	0.85	0.570	0.49	1.48	0.81	0.45	1.48	0.867
Chronic fluoxetine	0.66	0.463	0.21	2.02	0.69	0.22	2.14	0.867
Chronic fluvoxamine	1.00	1.000	0.16	6.35	0.83	0.11	6.10	0.964
Chronic inhaled ciclesonide	1.11	0.772	0.55	2.24	1.26	0.60	2.66	0.867
Chronic cochicine	1.05	0.933	0.37	2.95	1.12	0.38	3.29	0.964
Chronic NSAID	0.89	0.391	0.69	1.16	0.85	0.65	1.12	0.734

Chronic famotidine	0.83	0.763	0.25	2.80	0.60	0.16	2.28	0.867
Chronic PPI	0.88	0.118	0.76	1.03	0.92	0.77	1.08	0.747
Acute HCQ	0.21	0.153	0.02	1.78	0.22	0.02	1.99	0.734
Acute doxycycline	1.26	0.581	0.55	2.86	1.48	0.62	3.54	0.861
Acute azithromycin	1.29	0.650	0.43	3.83	1.27	0.41	3.97	0.964

¶ Corrected for multiple testing using Benjamini-Hochberg approach

Abbreviation: angiotensin converting enzyme inhibitors (ACEi); angiotensin receptor blockers (ARBs); beta blockers (BBs); calcium channel blockers (CCBs); dipeptidyl peptidase (DPP)-4 inhibitor; glucagon-like peptide (GLP)-1; sodium-glucose cotransporter (SGLT)-2 inhibitor; non-selective monoamine reuptake inhibitors (NSMRI); selective serotonin reuptake inhibitor (SSRIs); nonsteroidal anti-inflammatory drugs (NSAIDs); proton-pump inhibitors (PPIs); hydroxychloroquine (HCQ)

Table S2 Conditional logistic regression analysis – crude and adjusted odds ratios (OR) and 95% confidence interval (lower and upper limit) of candidate drugs for repurposing for prevention of COVID-19 hospitalization

Candidate drug	Crude OR	p value	Lower limit	Upper limit	Adjusted OR	Lower limit	Upper limit	p value ¶
Chronic ACEi	0.38	0.362	0.05	3.00	0.59	0.03	11.23	0.913
Chronic ARB	3.00	0.091	0.87	10.36	9.46	0.76	117.37	0.896
Chronic BB	1.77	0.280	0.64	4.88	2.48	0.39	15.84	0.896
Chronic CCB	2.32	0.138	0.78	6.88	1.30	0.13	13.14	0.913
Chronic diuretics	1.54	0.500	0.44	5.35	2.17	0.27	17.29	0.896
Chronic statin	0.70	0.521	0.24	2.05	0.96	0.12	7.79	0.970
Chronic NSMRI	3.87	0.349	0.24	63.34	3.16	0.07	135.69	0.896
Chronic SSRI	1.13	0.871	0.26	4.96	9.22	0.20	427.56	0.896
Chronic Antipsychotics	1.85	0.621	0.16	20.99	0.17	0.00	15.62	0.896
Chronic NSAID	0.43	0.444	0.05	3.68	0.74	0.06	9.55	0.913
Chronic PPI	0.82	0.666	0.34	1.99	0.53	0.11	2.53	0.896

¶ Corrected for multiple testing using Benjamini-Hochberg approach

Abbreviation: angiotensin converting enzyme inhibitors (ACEi); angiotensin receptor blockers (ARBs); beta blockers (BBs); calcium channel blockers (CCBs); non-selective monoamine reuptake inhibitors (NSMRI); selective serotonin reuptake inhibitor (SSRIs); nonsteroidal anti-inflammatory drugs (NSAIDs); proton-pump inhibitors (PPIs)

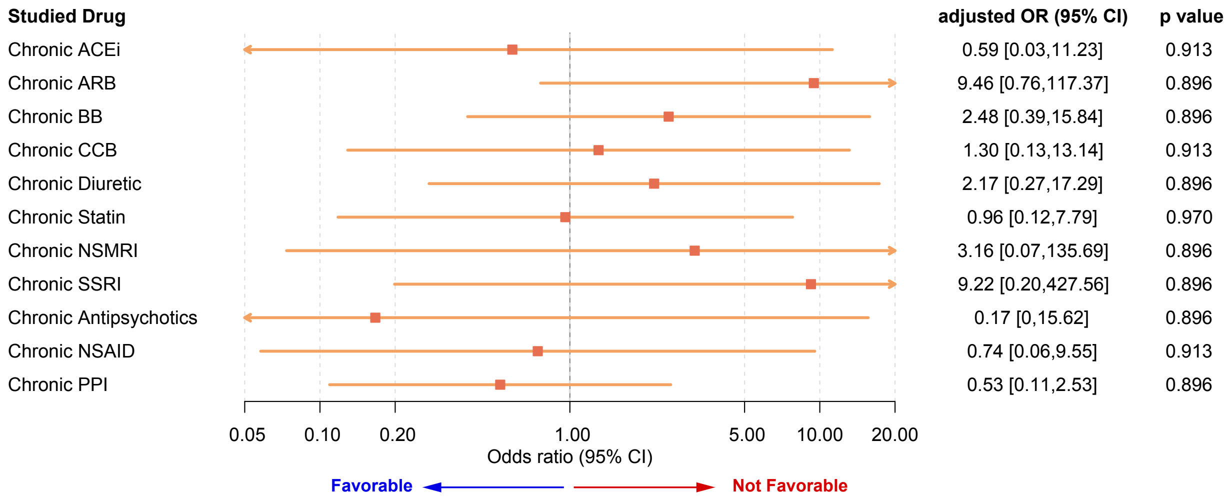

Figure S1 Forest plot of multivariable conditional logistic regression showing adjusted odds ratio (OR) for each candidate drug for the prevention of COVID-19 hospitalization

Abbreviation: angiotensin converting enzyme inhibitors (ACEi); angiotensin receptor blockers (ARBs); beta blockers (BBs); calcium channel blockers (CCBs); non-selective monoamine reuptake inhibitors (NSMRI); selective serotonin reuptake inhibitor (SSRIs); nonsteroidal anti-inflammatory drugs (NSAIDs); proton-pump inhibitors (PPIs)

Table S3 Conditional logistic regression analysis – crude and adjusted odds ratios (OR) and 95% confidence interval of candidate drugs for SARS-CoV-2 infection, stratified by SARS-CoV-2 variants

SARS-CoV-2 variant	Candidate drug	Adjusted OR	Lower limit	Upper limit	p value ¶
Original	Chronic ACEi	1.16	0.80	1.68	0.832
Original	Chronic ARB	0.79	0.51	1.24	0.705
Original	Chronic BB	0.95	0.67	1.36	0.899
Original	Chronic CCB	2.13	1.45	3.13	0.004
Original	Chronic diuretics	2.23	1.50	3.32	0.004
Original	Chronic statin	0.84	0.60	1.17	0.705
Original	Chronic metformin	4.31	1.91	9.69	0.009
Original	Chronic insulin	0.93	0.35	2.46	0.951
Original	Chronic aspirin	1.10	0.66	1.82	0.899
Original	Chronic NSMRI	1.51	0.80	2.84	0.670
Original	Chronic SSRI	0.52	0.29	0.93	0.179
Original	Chronic NSAID	0.58	0.36	0.93	0.174
Original	Chronic PPI	0.87	0.66	1.14	0.705
Alpha	Chronic ACEi	0.94	0.65	1.37	0.899
Alpha	Chronic ARB	0.94	0.65	1.36	0.899
Alpha	Chronic BB	1.13	0.82	1.54	0.832
Alpha	Chronic CCB	0.98	0.66	1.47	0.964
Alpha	Chronic diuretics	0.92	0.60	1.39	0.899
Alpha	Chronic statin	0.91	0.67	1.23	0.832
Alpha	Chronic metformin	0.91	0.40	2.07	0.909
Alpha	Chronic insulin	0.73	0.27	1.96	0.832
Alpha	Chronic aspirin	0.94	0.58	1.51	0.899
Alpha	Chronic NSMRI	0.98	0.52	1.83	0.964
Alpha	Chronic SSRI	0.90	0.52	1.56	0.899
Alpha	Chronic NSAID	0.69	0.46	1.04	0.365
Alpha	Chronic PPI	0.90	0.70	1.15	0.776
Delta	Chronic ACEi	2.05	0.99	4.25	0.291
Delta	Chronic ARB	0.75	0.29	1.96	0.840
Delta	Chronic BB	1.31	0.63	2.72	0.832
Delta	Chronic CCB	2.48	1.20	5.15	0.137
Delta	Chronic diuretics	3.10	1.35	7.10	0.105

Delta	Chronic statin	1.94	1.00	3.77	0.291
Delta	Chronic metformin	2.88	0.37	22.32	0.705
Delta	Chronic insulin	1.05	0.12	9.14	0.964
Delta	Chronic aspirin	1.07	0.39	2.97	0.951
Delta	Chronic NSMRI	3.07	0.48	19.64	0.670
Delta	Chronic SSRI	1.43	0.46	4.47	0.832
Delta	Chronic NSAID	0.25	0.05	1.17	0.365
Delta	Chronic PPI	0.49	0.26	0.91	0.174
Omicron BA 1/2	Chronic ACEi	0.96	0.71	1.31	0.899
Omicron BA 1/2	Chronic ARB	1.37	0.95	1.99	0.395
Omicron BA 1/2	Chronic BB	1.12	0.83	1.52	0.832
Omicron BA 1/2	Chronic CCB	0.96	0.69	1.33	0.899
Omicron BA 1/2	Chronic diuretics	0.79	0.57	1.09	0.531
Omicron BA 1/2	Chronic statin	1.17	0.91	1.51	0.670
Omicron BA 1/2	Chronic metformin	0.88	0.43	1.77	0.899
Omicron BA 1/2	Chronic insulin	0.84	0.34	2.10	0.899
Omicron BA 1/2	Chronic aspirin	1.79	1.15	2.78	0.105
Omicron BA 1/2	Chronic NSMRI	0.69	0.38	1.25	0.670
Omicron BA 1/2	Chronic SSRI	0.73	0.45	1.21	0.670
Omicron BA 1/2	Chronic NSAID	1.11	0.76	1.63	0.846
Omicron BA 1/2	Chronic PPI	1.04	0.83	1.30	0.899
Omicron BA 4/5	Chronic ACEi	0.67	0.30	1.51	0.705
Omicron BA 4/5	Chronic ARB	1.63	0.59	4.53	0.709
Omicron BA 4/5	Chronic BB	2.22	0.83	5.92	0.427
Omicron BA 4/5	Chronic CCB	1.71	0.59	4.97	0.705
Omicron BA 4/5	Chronic diuretics	2.20	0.86	5.65	0.408
Omicron BA 4/5	Chronic statin	3.42	1.35	8.64	0.105
Omicron BA 4/5	Chronic metformin	1.73	0.15	19.44	0.899
Omicron BA 4/5	Chronic insulin	2.18	0.12	39.74	0.864
Omicron BA 4/5	Chronic aspirin	1.82	0.54	6.14	0.705
Omicron BA 4/5	Chronic NSMRI	0.61	0.14	2.70	0.832
Omicron BA 4/5	Chronic SSRI	1.67	0.33	8.35	0.832
Omicron BA 4/5	Chronic NSAID	1.75	0.59	5.24	0.705
Omicron BA 4/5	Chronic PPI	1.02	0.53	1.95	0.964

¶ Corrected for multiple testing using Benjamini-Hochberg approach

Abbreviation: angiotensin converting enzyme inhibitors (ACEi); angiotensin receptor blockers (ARBs); beta blockers (BBs); calcium channel blockers (CCBs); non-selective monoamine reuptake inhibitors (NSMRI); selective serotonin reuptake inhibitor (SSRIs); nonsteroidal anti-inflammatory drugs (NSAIDs); proton-pump inhibitors (PPIs)

Table S4 Conditional logistic regression analysis – crude and adjusted odds ratios (OR) and 95% confidence interval of candidate drugs for SARS-CoV-2 infection, stratified by vaccination status

Candidate drug	Vaccination status	Adjusted OR	Lower limit	Upper limit
Chronic ACEi	Unvaccinated	0.93	0.66	1.32
Chronic ACEi	Vaccinated, <3 months	1.11	0.70	1.75
Chronic ACEi	Vaccinated, 3-6 months	0.94	0.61	1.45
Chronic ACEi	Vaccinated, >6 months	1.24	0.51	3.03
Chronic ARB	Unvaccinated	0.92	0.64	1.33
Chronic ARB	Vaccinated, <3 months	1.07	0.61	1.87
Chronic ARB	Vaccinated, 3-6 months	1.44	0.84	2.45
Chronic ARB	Vaccinated, >6 months	0.63	0.21	1.90
Chronic BB	Unvaccinated	1.21	0.90	1.64
Chronic BB	Vaccinated, <3 months	0.98	0.63	1.54
Chronic BB	Vaccinated, 3-6 months	1.31	0.86	1.99
Chronic BB	Vaccinated, >6 months	1.48	0.50	4.42
Chronic CCB	Unvaccinated	1.45	1.03	2.04
Chronic CCB	Vaccinated, <3 months	0.64	0.37	1.12
Chronic CCB	Vaccinated, 3-6 months	1.42	0.89	2.27
Chronic CCB	Vaccinated, >6 months	1.12	0.35	3.62
Chronic diuretics	Unvaccinated	1.28	0.89	1.85
Chronic diuretics	Vaccinated, <3 months	0.89	0.54	1.47
Chronic diuretics	Vaccinated, 3-6 months	1.06	0.67	1.67
Chronic diuretics	Vaccinated, >6 months	5.47	1.11	26.82
Chronic statin	Unvaccinated	0.91	0.68	1.22
Chronic statin	Vaccinated, <3 months	1.13	0.79	1.64
Chronic statin	Vaccinated, 3-6 months	1.11	0.77	1.58
Chronic statin	Vaccinated, >6 months	1.58	0.68	3.67
Chronic metformin	Unvaccinated	1.51	0.80	2.86
Chronic metformin	Vaccinated, <3 months	1.37	0.64	2.92
Chronic metformin	Vaccinated, 3-6 months	0.97	0.44	2.14
Chronic metformin	Vaccinated, >6 months	1.20	0.27	5.29
Chronic insulin	Unvaccinated	0.90	0.36	2.29
Chronic insulin	Vaccinated, <3 months	1.24	0.42	3.67
Chronic insulin	Vaccinated, 3-6 months	0.82	0.19	3.60

Chronic insulin	Vaccinated, >6 months	/	/	/
Chronic aspirin	Unvaccinated	0.97	0.61	1.53
Chronic aspirin	Vaccinated, <3 months	1.71	0.91	3.19
Chronic aspirin	Vaccinated, 3-6 months	1.45	0.79	2.67
Chronic aspirin	Vaccinated, >6 months	1.01	0.25	4.14
Chronic NSMRI	Unvaccinated	1.16	0.68	1.97
Chronic NSMRI	Vaccinated, <3 months	1.28	0.48	3.39
Chronic NSMRI	Vaccinated, 3-6 months	0.90	0.40	2.05
Chronic NSMRI	Vaccinated, >6 months	0.75	0.15	3.69
Chronic SSRI	Unvaccinated	0.76	0.48	1.20
Chronic SSRI	Vaccinated, <3 months	0.46	0.17	1.30
Chronic SSRI	Vaccinated, 3-6 months	1.12	0.57	2.18
Chronic SSRI	Vaccinated, >6 months	0.50	0.19	1.29
Chronic NSAID	Unvaccinated	0.63	0.42	0.94
Chronic NSAID	Vaccinated, <3 months	1.22	0.62	2.41
Chronic NSAID	Vaccinated, 3-6 months	1.19	0.70	2.03
Chronic NSAID	Vaccinated, >6 months	0.88	0.29	2.70
Chronic PPI	Unvaccinated	0.85	0.67	1.08
Chronic PPI	Vaccinated, <3 months	0.92	0.65	1.30
Chronic PPI	Vaccinated, 3-6 months	1.07	0.78	1.46
Chronic PPI	Vaccinated, >6 months	0.70	0.32	1.54

Abbreviation: angiotensin converting enzyme inhibitors (ACEi); angiotensin receptor blockers (ARBs); beta blockers (BBs); calcium channel blockers (CCBs); non-selective monoamine reuptake inhibitors (NSMRI); selective serotonin reuptake inhibitor (SSRIs); nonsteroidal anti-inflammatory drugs (NSAIDs); proton-pump inhibitors (PPIs)

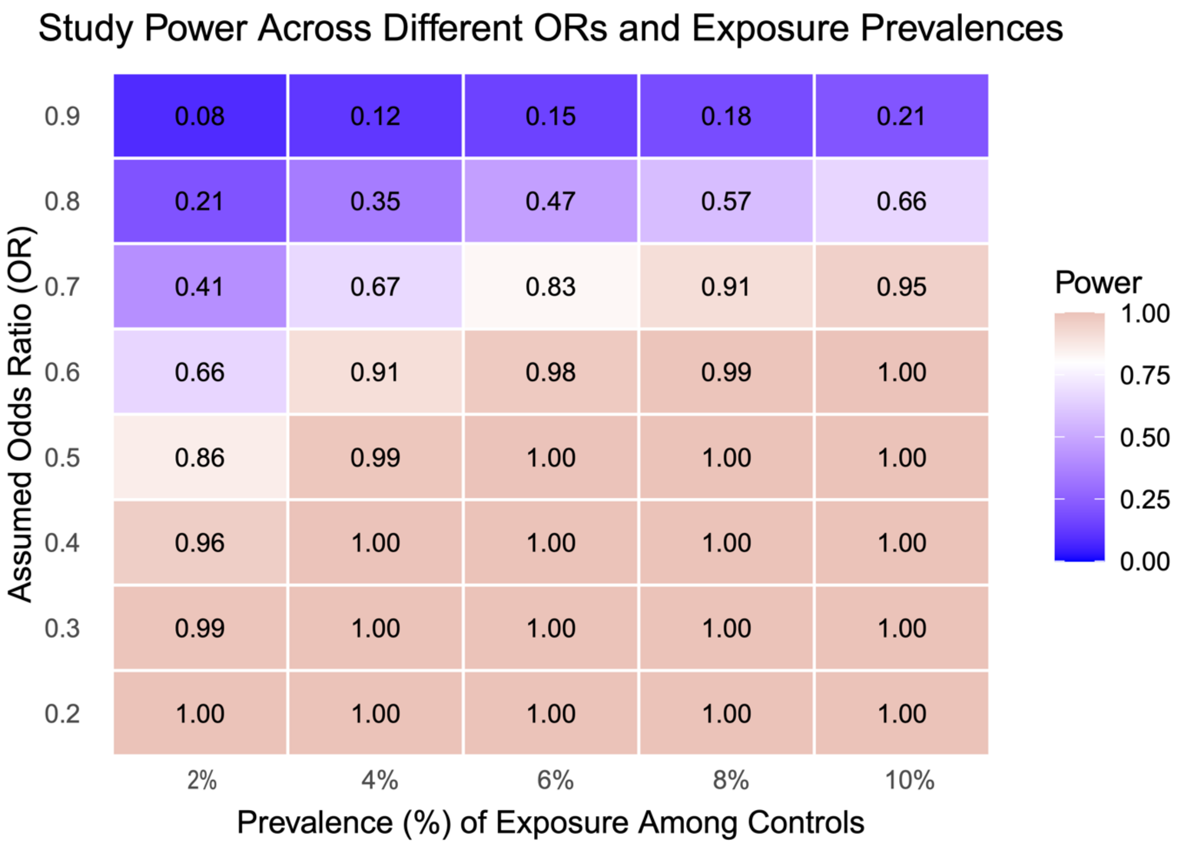

Figure S2 Heat plot illustrating the study power at different levels of odds ratios and exposure prevalence

There were 2019 cases and 4089 matched controls. The significance level was 0.05 (two-sided). The center point of the heat plot was 0.80, which is an acceptable level of statistical power.

Table S5 List of candidate drugs of interest and corresponding ATC code

**Medication**	**ATC code**	**Medication**	**ATC code**
**Chronic drugs**			
Drugs for cardiovascular system			
Angiotensin-converting-enzyme inhibitors (ACEi)	C09AA	Angiotensin receptor blockers (ARBs)	C09CA
Beta-blocking agents	C07A	Calcium channel blockers (CCB)	C08
Diuretics	C03	Statin	C10AA
Antivirals			
Lopinavir/Ritonavir (LPV/r)	J05AR10	tenofovir disoproxil/emtricitabine (TDF/FTC)	J05AR03
Ribavirin	J05AP01		
Antidiabetic drugs			
Metformin	A10BA02	Sulfonylureas	A10BB
Thiazolidinediones	A10BG	DPP-4 inhibitors	A10BH
GLP-1 analogues	A10BJ	SGLT2 inhibitors	A10BK
Insulin	A10A		
Antithrombotic agents			
Aspirin as antithrombotic agent	B01AC06	Warfarin	B01AA03
Heparin group	B01AB		
Antipsychotics and antidepressants			
Non-selective monoamine reuptake inhibitors	N06AA	Selective serotonin reuptake inhibitors (SSRIs)	N06AB
Monoamine oxidase inhibitors (MAOIs), non-selective	N06AF	Monoamine oxidase inhibitors (MAOIs)	N06AG
Other antidepressants	N06AX	Antipsychotics	N05A
Fluoxetine	N06AB03	Fluvoxamine	N06AB08
Others			
Ciclesonide (inhalation)	R03BA08	Ciclesonide (nasal)	R01AD13
Colchicine	M04AC01	Non-steroidal anti-inflammatory drugs (NSAIDs)	M01A
Famotidine	A02BA03	Proton pump inhibitors (PPIs)	A02BC
Camostat	B02AB04		
**Acute drugs**			

Antimalarials and antiparasitic			
Hydroxychloroquine	P01BA02	Chloroquine	P01BA01
Ivermectin	P02CF01		
Antivirals and antibacterial			
Azithromycin	J01FA10	Umifenovir	J05AX13
Doxycycline	J01AA02	Favipiravir	J05AX27
Others			
Melatonin	N05CH01		

These candidate drugs were selected from our previous systematic review: Repurposed drug studies on the primary prevention of SARS-CoV-2 infection during the pandemic: systematic review and meta-analysis

Table S6 Outcome definitions based on the Lifelines COVID-19 cohort questions

Outcome	Questions in Lifelines COVID-19 cohort	Required answer
SARS-CoV-2 infection present	Was the result of (one of your) test(s) for coronavirus (COVID-19) at the GGD positive?	Yes
	Was the result of (one of your) test(s) for COVID-19 positive?<br>Were the test(s) performed by the GGD?	Both yes
Hospitalization for SARS-CoV-2 infection	Have you been hospitalized for a Covid-19 infection?	Yes
ICU admission for SARS-CoV-2 infection	Were you in the intensive care unit of the hospital?	Yes

Table S7 Definition of covariates

Variable	Definition	Classification	Data source
Age	Age at index date (see methods section)	Continuous	Lifelines
Sex	Male or female	Categorical	IADB.nl
Ethnicity	- White/eastern and western European<br>- White/Mediterranean or Arabic<br>- Black/Negroid<br>- Asian<br>- Other	Categorical	Lifelines
Educational level	- Low (no education; primary education; lower or secondary vocational education; junior general secondary education)<br>- Middle (secondary vocational education or work-based learning pathway; senior general secondary education or pre-university secondary education)<br>- High (higher vocational education; university education)<br>- Other<br>Choose the assessment closest to the index date.	Categorical	Lifelines
Marital status	- Single<br>- Registered partnership<br>- Married<br>- Other<br>Choose the assessment closest to the index date.	Categorical	Lifelines
Vaccination status at index date	- Unvaccinated (not received any dose of COVID-19 vaccines as of the index date or the date of vaccination is < 14 days prior to the index date)	Categorical	CIMS

	- Vaccinated, <3 months before index date (received at least one dose of COVID-19 vaccines (Spikevax [Moderna], Comirnaty [BioNTech/Pfizer], Vaxzevria [AstraZeneca]), and time since last dose at index date is between 14 days and 3 months)<br><br>- Vaccinated, 3-6 months before index date (received at least one dose of COVID-19 vaccines (Spikevax [Moderna], Comirnaty [BioNTech/Pfizer], Vaxzevria [AstraZeneca]), and time since last dose at index date is between 3 and 6 months)<br><br>- Vaccinated, >6 months (received at least one dose of COVID-19 vaccines (Spikevax [Moderna], Comirnaty [BioNTech/Pfizer], Vaxzevria [AstraZeneca]), and time since last dose at index date is more than 6 months)		
Health-cautiousness behavior	- Cautious (go for test when mild symptoms happen)<br>- Not cautious (do not go for test when mild symptoms happen)	Categorical	Lifelines COVID-19 questionnaire
Current smoking	Yes or no.<br>Choose the assessment closest to the index date.	Categorical	Lifelines
BMI	- Underweight (BMI < 18.5)<br>- Normal (BMI 18.5-24.9)<br>- Overweight (BMI 25-29.9)<br>- Obese (BMI ≥ 30)<br>Choose the assessment closest to the index date.	Categorical	Lifelines
Alcohol consumption	- Low (< 1 unit/glasses per week)<br>- Moderate (1-8 units/glasses per week)	Categorical	Lifelines

	- High (≥ 8 units/glasses per week)<br>Choose the assessment closest to the index date.		
Comorbidity at index date	If a person has a drug prescription in IADB.nl corresponding to the disease or self-reports in Lifelines, then yes	Categorical	IADB.nl; Lifelines

Lifelines Corona Research Initiative

H. Marike Boezen[1], Jochen O. Mierau[2,3,9], H. Lude Franke[4], Jackie Dekens[4,6], Patrick Deelen[4], Pauline Lanting[4], Judith M. Vonk[1], Ilja Nolte[1], Anil P.S. Ori[4,5], Annique Claringbould[4], Floranne Boulogne[4], Marjolein X.L. Dijkema[4], Henry H. Wiersma[4], Robert Warmerdam[4], Soesma A. Jankipersadsing[4], Irene van Blokland[4,7], Geertruida H. de Bock[1], Judith GM Rosmalen[5,8], Cisca Wijmenga[4].

[1] Department of Epidemiology, University of Groningen, University Medical Center Groningen, Groningen, The Netherlands

[2] Department of Economics, Econometrics & Finance, Faculty of Economics and Business, University of Groningen, Groningen, The Netherlands

[3] Lifelines Cohort Study and Biobank, Groningen, The Netherlands

[4] Department of Genetics, University of Groningen, University Medical Center Groningen, Groningen, The Netherlands

[5] Department of Psychiatry, University of Groningen, University Medical Center Groningen, Groningen, The Netherlands

[6] Center of Development and Innovation, University of Groningen, University Medical Center Groningen, Groningen, The Netherlands

[7] Department of Cardiology, University of Groningen, University Medical Center Groningen, Groningen, The Netherlands

[8] Department of Internal Medicine, University of Groningen, University Medical Center Groningen, Groningen, The Netherlands

[9] Team Strategy & External Relations, University of Groningen, University Medical Center Groningen, the Netherlands
